# The influencing factor model and empirical research of TikTok charity live streaming impact users’ online charitable donation

**DOI:** 10.1371/journal.pone.0294186

**Published:** 2023-11-13

**Authors:** Yujing Shi, Chenyang Wu

**Affiliations:** School of Journalism & Communication, Chongqing University, Chongqing, China; UTM Skudai: Universiti Teknologi Malaysia, MALAYSIA

## Abstract

The "live streaming + charity" model is a new model for China’s philanthropy, accelerating the new development of China’s philanthropy, but there is still a relative paucity of research in the academic community on how charity live streaming affects online charitable donations. In this sense, this study aims to identify the construction of a model of the factors influencing charity live streaming on online charitable donations. This study selected TikTok Live, based on the UTAUT model, combining perceived risk and perceived interactivity, recovered 607 valid questionnaires, and concluded and structural equation modeling to construct an influence factor model to analyze their correlation. The results show that users’ performance expectancy, effort expectancy, perceived interactivity, facilitating conditions, and social influence are significantly positively correlated with online charitable donations, and perceived risk does not negatively affect users’ intentions to make online charitable donations. Our findings can provide a basis for live-streaming platforms and relevant social organizations and government departments to develop charity communication strategies.

## Introduction

With the rapid development of Internet technology, online charity has become the most important form and the most distinctive feature of modern charity. The Internet has enriched the channels of public participation in charity, and also enhanced its accessibility and convenience. The boom in online live streaming coincides with growth in online charitable giving. Online live streaming, especially gaming-related streaming, also generates significant charitable donations. This type of activity is particularly common on the Twitch platform, especially at a time when traditional fundraising campaigns run into various sociopolitical obstacles and struggle to attract younger generation [1]. An independent survey showed that independent online content creators raised more than $20 million for charity in 2018, of which $12 million came specifically from game-related channels and content [2]. More and more researchers have begun to study this model of charitable giving, trying to understand why people donate to streaming media, why organizations use live streaming platforms to raise funds, and how fundraising through live streaming differs from traditional fundraising methods [3].

Although Twitch is very popular in Europe and the United States, it is not as popular as TikTok Live in China, because of the different social context. A large number of studies have shown that charitable organizations use Twitch to raise funds mainly for public relations or marketing purposes [3]. However, in China, the purpose of fundraising is Promote the development of social welfare undertakings. Charity live streaming programs are often jointly planned and led by the government and charitable organizations, which indicates these programs are subject to the supervision of relevant government departments and the TikTok Live platform. Similar to the characteristics of raising funds through Twitch, compared with other methods of raising funds, raising funds through TikTok Live has the lowest cost and can create a wider and more diverse potential donor group. Ability to bring new donors and donation resources to charitable organizations.

In China, the 2020 China Charity Donation Report [4] conducted by China Charity Alliance shows that Charity organizations in China raised more than 8.2 billion yuan through 20 online fundraising platforms in 2020. More than 10 billion people participated in online donations, highlighting the vitality of the “Internet + Charity” mode. What needs to be concerned is the emergence of TikTok Live as a new charitable fundraising platform, and its technological characteristics for fundraising dissemination to create more possibilities. Therefore, research on the influencing factors of individual online donation behavior has become an important topic.

Traditional studies of charitable donation intentions have mostly been conducted at the approach of psychological mechanisms, examining the relationship between donors and recipients or donors’ perceptions of their donation behaviors, and thus these studies have covered a variety of domains, Ranganathan and Henley had concluded these include: attitudes toward charitable organizations, altruism, religious beliefs, participation, donor characteristics, and size of requests. Studies have also focused on individual donor characteristics and norms. For instance, Majumdar and Bose [5] find the impact of discourse in charitable donation on the effectiveness of giving from a narratological path. Chen et al. [6] combined the Theory of Planned Behavior, Norm Activation Theory, and Social Presence Theory and developed a new integrative framework for measuring donation intentions. Lee and Park [7] indicate philanthropy as a stewardship activity is examined in terms of how charitable donation organizations should attract potential donors in similar philanthropic programs and how to achieve the desired goals of crowdfunding programs. At present, researchers are still unclear about TikTok Live’s fundraising mechanism.

However, live streaming, as a new technology for charity organizations to attract individuals to participate in donating, has not yet received much attention. Previous research has examined the online work of nonprofits [8, 9], and systems of computer-mediated charitable giving [10–12]. Some scholarships have also examined the practice of online live streaming subcommunities [13–15] and viewer motivations and interaction with live streams [16–18]. But they tend to focus on social media, such as Facebook [19] and Instagram [20], rather than live stream media. In all, there is a lack of techno-logical or techno-motivational perspectives to inquire about the impact of users’ cognition and participation in charity live streaming and making charitable donations.

### Theoretical background and hypothesis

#### Charity live streaming

Mittal and Wohn [3] indicate that charity live streaming is a novel and increasingly popular form of fundraising where content creators stream content during a fixed period to raise money and awareness, and many charitable live streaming involve people playing games for a prolonged period. However, there are some differences between charity live streaming in China and the West. Unlike Twitch, popularized in recent years, which raises money for charity by playing and streaming games on live apps or platforms, charity live streaming generally be held by the government, NGO, or caring enterprise in China, and the requirements for charitable fundraising are very strict. For instance, the content of charity live streaming is usually grander, such as organized song and dance performances, public welfare assistance to farmers, poverty alleviation live streaming, and so on. If streamers initiate charity live streaming on the platform, they need to be registered in the civil affairs department, submit legal registration certificates and proofs, and then authenticate them according to the regulations; if other streamers such as the government and the media initiate charitable fundraising on the platform, they need to hold the relevant qualifications, or joint with qualified organizations [21].

Charity live streaming has the following characteristics. First, the audience participation is broad. Studies have shown that whether it is a charity live streaming activity led by enterprises or public welfare organizations, the most obvious characteristic of its communication effect is the wide audience participation. This is mainly reflected in the strong audience participation of online live streaming, the online live streaming has a playback function, and the audience can watch it anytime and anywhere. Public welfare concepts can be quickly spread, public welfare forms are more diversified, and public welfare consensus has been formed to the greatest extent. Secondly, charity live streaming has a certain degree of transparency. Charity activities can be fully presented to the audience in the form of live streaming. In the live broadcast room, you can see the introduction of charity projects, participation methods, number of participants, and the amount of donated funds and items.

#### Charitable donation in charity live streaming

Online charity donation refers to the behavior of relying on the Internet to make donations online. It has been widely used for its broad participation, timely diversity, and convenience. In western, donation in live-stream engagement is financial donation to the streamer (via PayPal) or charities, the donator is usually thanked personally by the streamer, celebrated in the chat room, and may have their name displayed on the stream as the “Top Donator” or a “Recent Donator” [22]. However, in China, there are two ways for users to make charitable donations in charity live streaming (via TikTok Live in China).

First is virtual gifting. In the live room, users can recharge through the platform to buy virtual items gifted to the streamer to show their support, different items corresponding to different amounts of coupons, the streamer will be able to use the coupons accumulated in each live stream to exchange for RMB, to achieve the donation. For example, the Tianjin Song and Dance Drama Theater National Orchestra’s young suona musician Mingfei Zhai, used the income from the live streaming to give back to the community. During the 2021 Henan Province’s Rainstorm Disaster, she did a charity live streaming on TikTok, and donated all her gifted income, more than 2,300 yuan, to the Henan Provincial Charity Federation [23].

Second is live commerce. Streamers can not only earn commerce commissions through live commerce and get charity money, but also directly buttress the helping objects to achieve the effect of direct donations, and even buttress the cooperative enterprises to achieve the effect of indirect donations. For example, on April 8, 2020, TikTok joint Hubei Provincial Internet Information Office, Hubei Provincial Department of Commerce, Hubei Provincial Department of Agriculture and Rural Affairs initiated the "TikTok help Hubei Province Recovery Plan", Wuhan Vice Mayor Li Qiang introduced Wuhan’s economic reboot and the resumption of work and production to many citizens, and promote some well-known Hubei enterprises’ products. As of May 21, 2020, TikTok has helped 13 cities bring goods for 234 million yuan, with 4.72 million pieces of hot-selling specialties [24].

Through literature review, it was found that there is currently a lack of research on the technical effects of charity live streaming. Most of the perspectives of relevant papers are discussions in the fields of charity advertising, charity event videos on Twitter or Facebook, and game live streaming fundraising. The research methods and research theories are mostly derived from marketing or public administration academics, which lack an integrated perspective.

#### The UTAUT model

**Fig 1:**
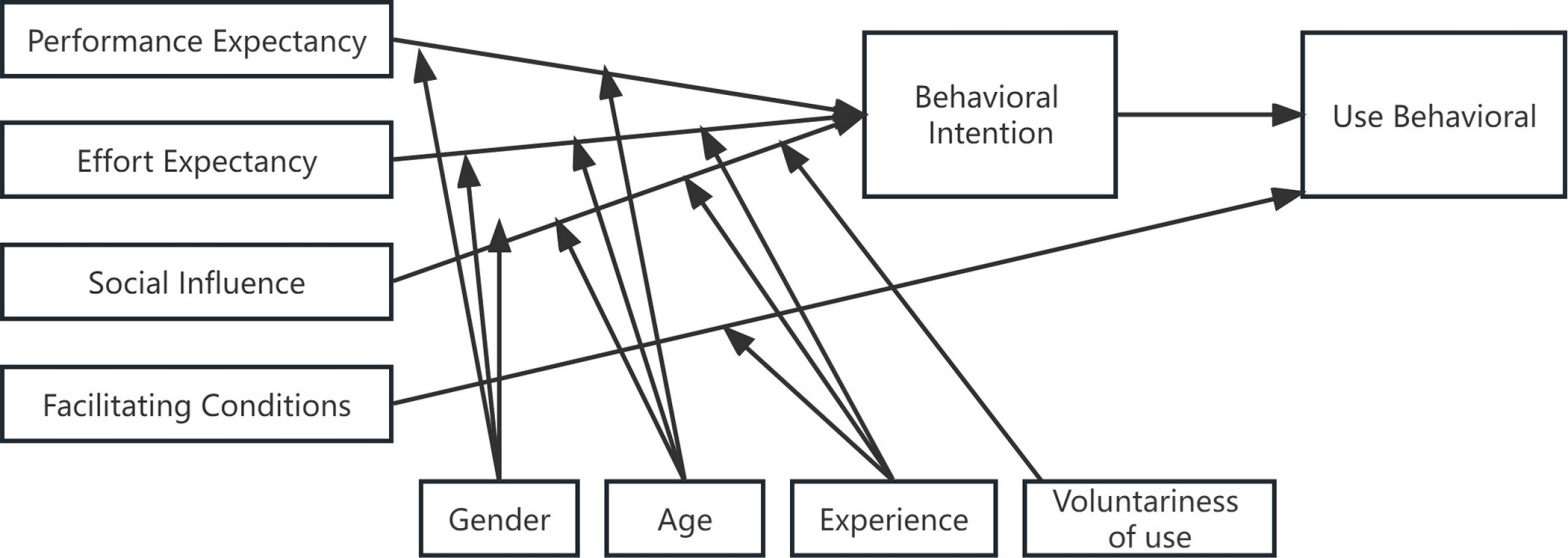
The UTAUT model.

The UTAUT model (Unified Theory of Acceptance and Use of Technology) is proposed by Venkatesh et al. [25] based on the integration of eight major technology acceptance theories and behavioral intention theories: the Theory of Rational Behavior (TRA), the Technology Acceptance Model (TAM), the Technology Acceptance Augmentation Model (TAM2), the Motivation Model (MM), the Theory of Planned Behavior (TPB), the Integrated TAM-TCP (C-TAM-TCP), the Model of PC Use (MPCU), the Diffusion of Innovation Theory (IDT) and Social Cognitive Theory (SCT), a comprehensive model (Unified Theory of Acceptance and Use of Technology) based on eight major theories of technology acceptance and behavioral intention. The model has an explanatory power of up to 70% for user behavioral intention and technology acceptance and is considered by many scholars to be an effective measurement tool. The UTAUT model has four core dimensions including performance expectancy, effort expectancy, social influence, and facilitating conditions. In addition, the model has four moderating variables, including gender, age, voluntariness, and experience. These four core variables have a direct effect on individual behavior intention, while the four moderating variables have an indirect effect, and ultimately have an effect on individual use behavior (Fig 1).

For this study, the UTAUT model, as a theoretical model that explains user behavioral intention and technology acceptance, can work well in our model construction. The UTAUT model is common in various information technology adoption studies, and its application areas involve information systems, mobile services, knowledge communities, online shopping, social media, and so on. Its four core dimensions have good explanatory ability and predictive ability. For live streaming, a small amount of literature has verified the explanatory ability of the UTAUT model, for example, Xiwei et al. [26] find that the performance expectancy, effort expectancy, and social influence in the UTAUT model have a significant positive impact on users’ live streaming APP usage behavior, and Ziyi [27] finds that the performance expectancy and social influence have a significant positive impact on the users’ watching of live streaming to help streamers to buy their products. It is worth noting that the UTAUT model changes its research variables and moderating variables differently in different technological and social environments, which in turn produces different research results. As Davis [28], also Taylor and Todd [29] suggest, in studies of acceptance behavior for different technologies, scholars can adjust the model based on technological and social contexts, or introduce more relevant factors from other theories as variables. As a new technology of charity communication, charity live streaming and online charitable donations expand the communication channels but also broaden the path of charitable donations, which is different from traditional charitable donations. Therefore, the online charitable donation behavior generated when users participate in the charity live streaming may be able to be explained by the UTAUT model to a certain extent.

Based on the UTAUT model, this study combines the characteristics of TikTok charity live streaming with performance expectancy, effort expectancy, social influence, and facilitating conditions as the four core influencing factors for analyzing users’ participation in online charitable donations through charity live streaming, and at the same time draws on variables involved in perceived risk and perceived interactivity in the theory of consumer behavior, to make it more suitable for targeting the research object. In addition, an online charitable donation through TikTok charitable live streaming is a new thing, charitable donations and communication technology are being gradually understood and accepted by China’s netizens, but the current use of the experience is still relatively limited, and not sufficient to measure, as users can freely choose to donate or not, there is no pressure from the external social forces of compulsory use, so the influence of voluntary role is also limited. Therefore, this study does not specifically consider the moderating effect of experience and voluntariness and proposes six influencing factors: performance expectancy, effort expectancy, social influence, facilitating conditions, perceived risk, and perceived interactivity, and puts forward six hypotheses based on this.

Performance expectancy consists of perceived usefulness, external incentives, job fit, relative advantage, and outcome expectations, and is designed to measure the performance enhancement that users perceive the technology used to bring to their work and learning [30]. Online charity has been shown to have the ability to provide quality information services, easy and efficient operational processes, and contribute to public service actions, so we retain this variable [31]. Based on previous introduction of TikTok Live in China, it is obvious that charity live streaming on TikTok Live has the ability of providing charity information and donation channels which donators need, thus, in this study, performance expectancy refers to the extent to which users believe in the efficacy of charitable giving by participating in online charitable donations through TikTok charity live streaming, so the hypothesis is:

H1: There is a significant positive correlation between users’ performance expectancy of participating in online charitable donations through TikTok charity live streaming and their online charitable donation intention.

Effort expectancy includes perceived ease of use, complexity, and ease of use, which aims to measure the degree of difficulty in using the technology as perceived by users [29]. The online charity has the characteristics of a simple donation procedure and low threshold, especially in charity live streaming, the donation methods are easy to access, such as virtual gifting, live shopping and direct live donations, which may make users feel more ease to donate, and resulting donation behavior, so the hypothesis is:

H2: There is a significant positive correlation between users’ effort expectancy to participate in online charitable donations through TikTok charity live streaming and their intentions to make online charitable donations.

Social influence includes subjective norms, social factors, and social image, and aims to measure the extent to which users are influenced by the social groups around them in the process of adopting technology [32]. In the process of the diffusion of new technologies, interpersonal communication channels have been proven to be more helpful in persuading audiences to adopt new technologies [33]. Thus, in the process of users choosing to participate in charitable donations through TikTok charity live streaming, the surrounding social groups may have an impact on their donation intentions and behaviors, so the hypothesis is:

H3: There is a significant positive correlation between social influence and users’ intention to participate in online charitable donations through TikTok charity live streaming.

Facilitating conditions consists of behavioral control perceptions, facilitating condition, and compatibility, which aims to measure the extent to which users believe that existing organizational and technological structures can support technology use [32]. On TikTok Live’s live streaming room, different interfaces show the charity information and donation cautions, with streamers’ introduction and interaction, also the comment area for users and live streaming assistants to communicate, and different payment methods authorized by the Chinese Government which users familiar with, such as WeChat Payment, Alipay Payment and TikTok Payment, may make users believe in the TikTok Live, then generate their intentions and behavior to donate through the charity live streaming room, so the hypothesis is:

H4: There is a significant positive relationship between facilitating conditions and users’ behavior of participating in online charitable donations through TikTok charity live streaming.

Perceived risk theory was first proposed by Bauer in 1967, Bauer defined perceived risk as "the combined perception of uncertainty of outcome and possible serious consequences" [34], then Peter and Ryan consider perceived risk to be people’s assessment of the potential loss associated with the purchase of a product or service, which is an inhibitor of purchasing behavior [35]. Jacoby and Kaplan, building on Bauer’s seminal work, categorized overall perceived risk into 5 types: financial risk, performance risk, physical risk, psychological risk, and social risk [36]. All these dimensions of perceived risk have been proved by scholars to have a significant impact on users’ intention to use information technology [37–39]. For instance, N. Salleh et al. [40] proves that perceived risk significantly affects personal information disclosure behavior on social networking sites. For online charitable donations through TikTok charity live streaming, the risk mostly comes from financial, privacy, and functional risks, such as the payment channel, privacy protection and, the authenticity risk of the charity program. Besides, from previous research on perceived risk and purchasing decisions, the stronger the perceived risk is, the weaker the purchasing intention is [41]. Thus, this study proposes the hypothesis:

H5: There is a significant negative correlation between perceived risk and users’ intention to participate in online charitable donations through TikTok charity live streaming.

Previous research has defined interactivity from four different perspectives, such as the characteristics of the technology, the process of exchanging information, the user’s perceptions of using the technology or experiencing the process, and a combination of these three perspectives [42]. In this study, perceived interactivity is defined as the extent to which users perceive their experience as a simulation of interpersonal interaction and feel that they are in the presence of social others [43]. Zhang et al. [44] confirm that information quality (credibility, usefulness, vividness) and interaction quality (responsiveness, instant interaction, empathy) are significantly related to consumers’ purchase intention on live e-commerce platforms based on social exchange theory. For TikTok Live, there are two main interactive methods which includes comment area and live streaming room, users can communicate with streamer and other users through both ways. This technology is consisted by two systems, which can be examined by two dimensions, user-user (human-computer or social interactivity) and user-system (machine interactivity) [45], with the former focusing on the interpersonal communication perspective and the response to the user’s posted content, and the latter emphasizing the technical features. Thus, we propose the hypothesis:

H6: There is a significant positive correlation between perceived interactivity on users’ participation in online charitable donations through TikTok charity live streaming.

**Fig 2:**
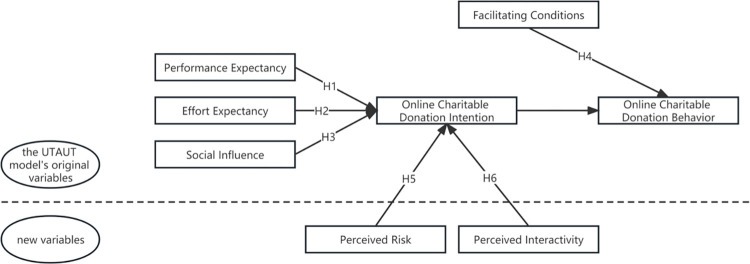
Schematic diagram of UTAUT model variable adjustment.

Based on the above theoretical foundation as well as the theoretical assumptions, we construct a conceptual model of the influencing factors of users’ participation in online charitable donations through TikTok charity live streaming, as shown in Fig 2:

## Methods

In terms of scale design, we designed a questionnaire (see S1 Appendix) on the influencing factors of participating in online charitable donations through TikTok charity live streaming concerning the research scales of relevant scholars (see S2 Appendix). The questionnaire is composed of 3 parts, including the introduction of the questionnaire, the survey of user demographic characteristics, and the scale part. The introduction part of the questionnaire briefly introduces the purpose of this study, the protection of users’ privacy, and explains to the users the definitions of TikTok charity live streaming and online charitable donations. The user demographic characteristics survey section includes information such as gender and age. The scale part includes the question items for measuring the variables, and during the initial period of questionnaire design, this study designed four questions for each variable, totaling 32 questions, and the question items were in the form of a 5-point Likert scale, including "Strongly Agree" "Agree" "Unsure" "Disagree" "Strongly Disagree".

A non-experimental design was used. Participants aged 18 years and over were recruited by advertising online on TikTok and direct online contact. Participation was voluntary. Data was gathered using an anonymous online survey over a two-week period during July, 2022. The survey took approximately 10 to 20 minutes to complete. Item sets were randomized in order to mitigate order effects and reduce the potential for response sets. All of the research procedures involving human participants were conducted according to the principles expressed in the Declaration of Helsinki. This study is a non-intervention study, does not interfere with the personal rights and privacy of regular participants, and does not increase the risk to participants. To fully protect the personal rights and privacy of the participants, before conducting the questionnaire survey, this research explains its purpose to the participants first and confirms the results of the study will be published as statistically analyzed data without any identifiable participant information, as this research also asks for the consent of the participants to ensure that they understand that their participation is voluntary and can be withdrawn at any time and participate anonymously. Thus, this study has been exempted from the ethics review by the ethics committee of Chongqing Nanan District Federation of Social Science Associations.

To meet the purpose of this study, we collected data through the Questionnaire Star (问卷星), which we disseminated via social networks. This platform has obtained the third-level information security certification recognized by Chinese authorities, and the ISO27001 information security system certification. To calculate the sample size, we used the SPSS25.0 and AMOS 24.0. Before distributing the questionnaires in large quantities, this study randomly distributed 50 questionnaires for pre-survey on July 10, 2022 –July 17, 2022, and corrected the questionnaires according to the results of reliability and validity tests, such as irrelevant items and unclearly stated items, to ensure the accuracy and reliability of the scale to the greatest extent possible. The corrected scale part of a total of 24 questions, to ensure the reliability and validity of the pre-survey questionnaire based on the questionnaire, and finally on July 21, 2022—July 28, 2022, formally distributed 650 questionnaires, the actual recovery of 607, of which 479 valid questionnaires, the effective questionnaire rate of 78.9%.

## Results

From the descriptive statistics of the survey sample (see Table 1), the proportion of men in the valid questionnaire accounted for 54.07%, and the proportion of women was 45.93%, with the proportion of men and women being relatively close, and the gender structure being more balanced. The age group of the respondents is the majority of young adults, with fewer middle-aged and elderly people. 18–29 years old and 40–49 years old account for a balanced proportion, the proportion of 30–39 years old is more than 40.29% of the total sample, while 50–59 years old accounts for 10.44%, and there are even fewer people of 60 years old and above, which is by the age structure of the Internet users.

**Table 1:** Descriptive statistical analysis of the sample population.

Statistical Quantity		Number of people	Proportion
**Gender**	Male	259	54.07%
	Female	220	45.93%
**Age**	18-29	127	26.51%
	30-39	193	40.29%
	40-49	108	22.55%
	50-59	50	10.44%
	60 and above	1	0.21%
**Monthly Salary**	≤¥2,000	71	14.82%
	¥2001-4000	297	62.00%
	¥4001-6000	102	21.29%
	¥6001-8000	8	1.67%
	>¥8000	1	0.21%
**Frequency**	frequently	195	40.71%
	occasionally	197	41.13%
	rarely	87	18.16%
**Donation Methods**	virtual gifting	194	40.50%
	live shopping	198	41.34%
	both	87	18.16%
**Donation Amount (live gifting)**	≤10¥	111	23.17%
	11-30¥	109	22.76%
	31-50¥	56	11.69%
	51-100¥	5	1.04%
	>100¥	0	0.00%
**Donation Amount (live shopping)**	≤200¥	195	40.71%
	201-500¥	80	16.70%
	501-800¥	8	1.67%
	801-1000¥	2	0.42%
	>1000¥	0	0.00%
**Payment Method**	WeChat Payment	413	86.22%
	Alipay Payment	319	66.60%
	Tiktok Payment	272	56.80%
	DOU Instalment	119	24.80%
**Access Channels**	the Home Page Recommendation Interface	282	58.87%
	the Same City Interface	318	66.40%
	the Follow Interface	212	44.26%
	the Search Interface	244	50.94%

### Reliability and validity

Before validating the hypothesized model, this study conducted a reliability and validity test through SPSS25.0, all indicators exceeded the recommended thresholds [46], which proves that the reliability and validity of the questionnaire met the criteria of factor analysis.

For the reliability test of sample data, the Cronbach’s α coefficient is usually used to measure the correlation of each question item, and the larger the coefficient is, the more reliable the result is. The test results (see Table 2) show that the Cronbach’s α coefficients of each variable are greater than 0.7, then it indicates that the measurement model has a good reliability coefficient and the internal consistency of the questionnaire is high, so it is possible to carry out subsequent analysis. For the validity test of the sample data, the KMO sample adequacy test and Bartlett’s sphericity test are needed to determine whether the scale is suitable for factor analysis. The test results (see Table 3) show that the KMO test coefficient of the scale part is 0.843, which is greater than the standard value of 0.6, and the Bartlett’s spherical test statistic is 8,513.42, with the corresponding probability significant level (Sig) of 0.000, which indicates that the correlation between the variables is stronger, which in turn can be used to conduct a validation factor analysis.

**Table 2:** Reliability test.

Variables	No. of questions	Cronbach’s α
**Performance Expectancy**	3	0.894
**Effort Expectancy**	3	0.884
**Social Influence**	3	0.890
**Facilitating Conditions**	2	0.886
**Perceived Risk**	3	0.872
**Perceived Interactivity**	3	0.884
**Donation Intention**	3	0.882
**Donation Behavior**	4	0.884

**Source:** The authors.

**Table 3:** KMO and bartlett sphericity test.

Testing Indicator	Statistical Value
**Kaiser-Meyer-Olkin Measurement of sampling adequacy**	0.843
**The chi-square approximation of the Bartlet’s spherical test**	8513.420
**df**	276
**significance**	0.000

### Validated factor analysis and model testing

**Fig 3:**
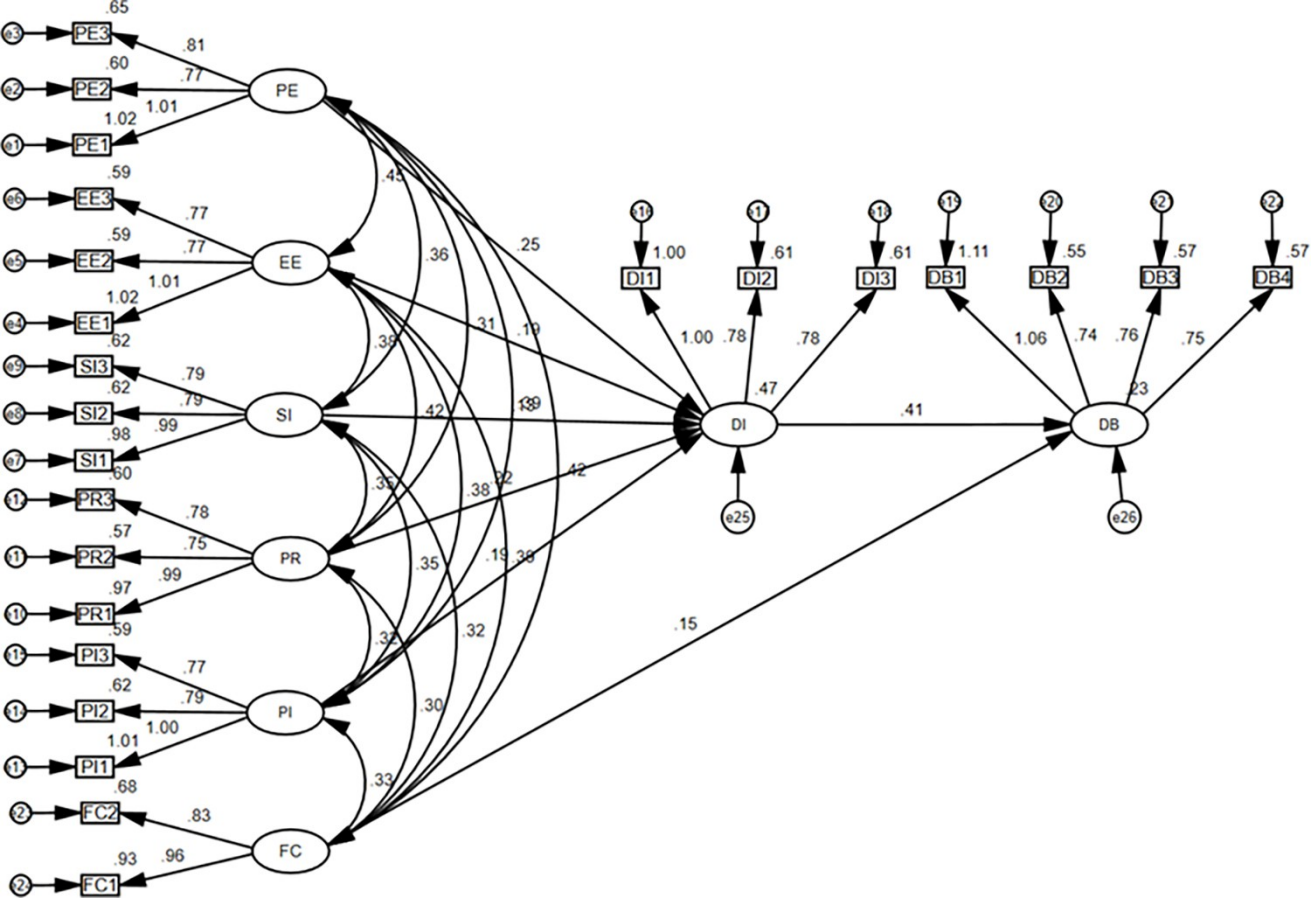
A model and standardized coefficients for participation in online charitable donation through TikTok charity live streaming.

In this study, validated factor analysis was conducted through structural equation modeling with the help of AMOS 24.0 and SPSS 25.0. The research variables are performance expectancy (PE1-PE3), effort expectancy (EE1-EE3), social influence (SI1-SI3), facilitating conditions (FC1-FC2), perceived risk (PR1-PR3), perceived interactivity (PI1-PI3), online charitable donation intention (DI1-DI3), and online charitable donation behavior (DB1-DB4). After importing the data, the structural equation model in Fig 3 is obtained, and the fitness indicators of the model are shown in Table 4.

**Table 4:** Structural equation fitness test.

Model fit coefficient	Statistical value	Optimal value	Fitting situation
**Chi-square**	294.409	-	
**Degrees of freedom**	230	-	
**Cardinality/degrees of freedom**	1.28	<3	good
**RMSEA**	0.024	<0.05	good
**GFI**	0.953	>0.9	good
**CFI**	0.992	>0.9	good
**IFI**	0.992	>0.9	good
**TLI**	0.991	>0.9	good
**NFI**	0.966	>0.9	good

According to the three criteria [47]: the standardized factor loading coefficients should be greater than 0. 5 and reach the significant level; the average variance extracted (AVE) should be above 0. 5; and the composite reliability (CR) should be above 0. 7. By calculating the individual paths of the performance expectancy, effort expectancy, social influence, facilitating conditions, perceived risk, perceived interactivity, online charitable donation intention, and online charitable donation behavior. The standardized factor loading coefficients were all above 0.5, the AVE are 0.747, 0.726, 0.742, 0.811, 0.714, 0.735, 0.700, 0.670, were all above 0.5, the CR is 0.897, 0.886, 0.895, 0.881, 0.891, 0.873, 0.888, were all above 0.7, indicating that each factor has good convergent validity and combined reliability.

### Parameter estimation and hypothesis testing

From the statistics in Table 5, it can be seen that the fitness index of the theoretical model of the study meets the criteria and the model fitness is good. Also from Table 6, it can be seen that the variables involved in H1, H2, H3, H4, and H6 have significant positive effects, so the hypothesis is valid. The perceived risk of H5 has a significant positive effect on users’ intention to make charitable donations (β = 0.216, p < 0.001), so the hypothesis is not valid.

**Table 5:** Validation factor analysis results.

Factors	Path	Loading Factor	AVE	CR
**Performance Expectancy**	PE1	1	0.750	0.899
	PE2	0.775		
	PE3	0.806		
**Effort Expectancy**	EE1	1	0.728	0.888
	EE2	0.771		
	EE3	0.768		
**Social Influence**	SI1	0.992	0.742	0.895
	SI2	0.787		
	SI3	0.789		
**Facilitating Conditions**	FC1	0.826	0.805	0.891
	FC2	0.963		
**Perceived Risk**	PC1	0.987	0.715	0.881
	PC2	0.754		
	PC3	0.776		
**Perceived Interactivity**	PI1	1	0.737	0.892
	PI2	0.788		
	PI3	0.768		
**Donation Intention**	DI1	1	0.740	0.894
	DI2	0.779		
	DI3	0.782		
**Donation Behavior**	DA1	1	0.673	0.890
	DA2	0.743		
	DA3	0.756		
	DA4	0.753		

**Table 6:** Model validation.

Path			Standardized load factor	Unstandardized load factor	S.E.	C.R.	P	Result
**Donation Intention**	<---	**Performance Expectancy**	0.249	0.248	0.04	6.267	***	valid
**Donation Intention**	<---	**Effort Expectancy**	0.186	0.187	0.041	4.532	***	valid
**Donation Intention**	<---	**Soical Influence**	0.131	0.138	0.041	3.363	***	valid
**Donation Intention**	<---	**Perceived Risk**	0.216	0.236	0.043	5.483	***	invalid
**Donation Intention**	<---	**Perceived Interactivity**	0.186	0.195	0.04	4.84	***	valid
**Donation Behavior**	<---	**Facilitating Conditions**	0.146	0.159	0.044	3.625	***	valid
**Donation Behavior**	<---	**Donation Intention**	0.406	0.413	0.04	10.263	***	valid

Note:*** indicates p<0.001

## Discussion

As can be seen from the data of the icons above, the hypotheses proposed in this study are supported by the data and the hypotheses are valid except for H5. The extrinsic latent variables that have the greatest impact on users’ participation in online charitable donations through live streaming for charity are performance expectancy (β = 0.249), followed by effort expectancy and perceived interactivity (β = 0.186), as well as facilitating conditions (β = 0.146) and social influence (β = 0.131). Perceived risk does not negatively affect users’ online charitable donation intention, and online charitable donation intention (β = 0.406) positively affects online charitable donation behavior. The discussion is analyzed as follows:

(1) The standardized estimate of performance expectancy on users’ intention is 0.249, and the standardized estimate of effort expectancy is 0.186, with a P-value of less than 0.001, indicating that performance expectancy and effort expectancy have a positive impact on users’ intention; and the standardized estimate of promotion conditions is 0.146, with a P-value of less than 0.001, indicating that the promotion conditions have a positive impact on users’ behavior. These three sets of data show that the process of legalization, professionalization, and civilization of China’s philanthropy is accelerating, the model of "live streaming + charity" has begun to bear fruit, and the acceptance of charity live streaming among enterprises, charity organizations, and the public is gradually increasing. The public increasingly agrees with the utility of charity live streaming and recognizes that live technology can help to achieve the purpose of their participation in charitable causes and improve the efficiency of charitable donations. This conclusion is consistent with Ziyi [27], the performance expectancy has the strongest positive influence on user behavior, the higher the actual benefits and values provided by the charity live streaming, the stronger the user’s behavioral intention, and the better the live streaming platform’s services, the stronger the user’s behavioral intention. This is also consistent with some studies on traditional charitable donation motivations, studies reveal that when people perceive that their contribution will not make a difference, they are less likely to give or leave a charitable bequest [48]. It can be seen that the "live streaming + charity" mode better meets user needs, which also suggests that the online live platform should start from the user needs, and constantly improve the charity live streaming and online charitable donations embedded in the technical logic and technical issues.

(2) The standardized estimate of the social influence on the user’s intention to participate in online charity donations through the charity live streaming is 0.131, P value less than 0.001, the data shows that the social impact on the user’s intention to have a positive impact. The results of this data show that users’ close people and media publicity have a certain impact on their intention to participate in online charitable donations through charity live streaming. This result is consistent with some studies that have identified factors which encourage users to donate online, specifically, the amount and likelihood of donating has been shown to increase when users know others who have donated [49]. This effect is more pronounced if acquaintances share similar characteristics, such as belonging to the same social group [50]. However, this result is different from some studies in China, for instance, Caiyun indicates that social influence has no significant effect on the use intention of social media APP [51], and Xiwei et al. also pointed out that social influence does not affect the use intention of live streaming APP users [26]. This slightly conflicting result reveals the gradual development of charity live streaming services in China and also reminds the Chinese Government and charitable organizations to pay attention to the role of social influence on charity live streaming, as it may bring more new donators to engage in online charitable donations.

(3) The standardized estimate of the impact of perceived risk on users’ intention to participate in online charitable donations through charity live streaming is 0.216, with a P value of less than 0.001, indicating that perceived risk has a positive impact on the intention to participate in online charitable donations. This result is different from other studies, especially in marketing filed, most of which found that the perceived risk of the research object has a significant negative impact on consumers’ purchase intention or impulse purchase intention, the stronger the consumers’ perception of the risks of the process and items of live commerce, the less likely they are to have an intention to purchase. However, in the charity live streaming environment, compared with the entertainment live streaming and commercial live streaming, it creates a sense of spatial presence and social presence that is unique, touching, or tragic, so it is conducive to the construction of the user’s sense of trust. Specifically, although there is a certain donation threshold in virtual gifting and live shopping, the amount of money is small, as giving a virtual “heart gift” only needs 1 cent yuan, live shopping is often inexpensive and has 7 days no reason to refund and other platform protection, the user will not constitute a large economic loss. This can be explained by some research findings that the smaller the donation amount, the easier it is for the audience to generate donation behavior, and the higher the donation amount is, the more difficult it is to generate donation behavior [52]. Zhao et al. also proved that reducing social vigilance leads to more charitable behavior, and propose that higher (vs. lower) perceived economic mobility promotes more charitable behavior of lower-income (vs. higher-income) consumers by reducing their social vigilance [53]. This result may cause some streamers benefit from this situation, such as deliberately sell misery to cheat money, which may corrupt the charity live streaming and online charitable donations to the credibility of the online. Therefore, the relevant government departments should introduce relevant charity live streaming management policies, live platforms should also strengthen the supervision of charity live streaming.

(4) The standardized estimate of the impact of perceived interactivity on users’ intention to participate in online charitable donations through charity live streaming is 0.131, with a P-value of less than 0.001, indicating that perceived interactivity has a positive impact on users’ intention to donate to online charities. This shows that live streaming technology can help generate the interactive ritual chain, create a value scene with social and emotional elements that are different from ordinary commercial live streaming form a ritual field of "promoting goodness", and encourage the public to participate in donations and charitable activities by bridging the vision, interest, and passion. This result is consistent with the research conclusions of some researchers on online donations, that is, the interactive elements of online live streaming provide audiences with immersive real-time social interaction and help enhance users’ willingness and actions to donate to charity [2]. Xu et al. also proved that interaction with streamers, group interactions and support for streamers can predict individual attitudes toward virtual gifting [54]. But we also need to pay attention to avoid excessive sensationalism brought about by the backlash. Therefore, the live streaming platform should strengthen the live regulation, and pay attention to the emotional atmosphere of the communication environment between the live streaming, to enhance the crowdfunding organizations, individual media literacy level.

(5) The standardized estimate of online charitable donation intention is 0.406, with a P-value of less than 0.001, indicating that online charitable donation intention has a significant positive impact on user behavior. This finding is consistent with the validation results of Venkatesh et al. [25], that is, the positive influence of intention on behavior. In the process of charity live streaming, users who are affected by multiple factors and have the intention to donate are still able to track the donated items because of blockchain technology after the live streaming, and the platform can take the initiative to continue the social relationship or donation relationship between crowdfunding organizations and individuals, which in turn leads to the occurrence of online charitable donation behavior. It should be noted that the impact of donation intention on donation behavior is the most important, in addition to the technical perspective provided by the UTAUT model, there should be other factors such as content, psychology, and other perspectives contained in other influences, the relevant government departments, live streaming platforms and charity organizations also need to cut from different perspectives, to do more exploratory factor analysis, to be able to understand the user’s donation intention formation and behavior transformation when watching charity live streaming.

## Conclusion

The theoretical contribution of this paper is to build a factor model of charity live streaming on user participation in online charitable donation based on the UTAUT model, and analyze the impact of China’s emerging Live APPs with charity live streaming and fundraising functions, represented by TikTok Live. At the same time, the two variables that reflect the characteristics of online live broadcasts, namely perceived interactivity and perceived risk, are used as the analysis variables of the model. Data analysis proves that user performance expectancy, effort expectancy, social influence, and facilitating condition have a positive impact on users’ willingness to donate, perceived interactivity has a positive impact on users’ willingness to donate, and perceived risk has a negative impact on users’ willingness to donate, but it is not significant for user donation behavior. Users’ online charitable donation intentions play a mediating variable role in users’ online charitable donation behavior. This article provides a new behavioral analysis model for the study of users’ charitable donations in charity live streaming.

The practical value of this paper lies in the analysis through questionnaires and structural equations. The results of empirical analysis indicate charity live streaming as a technology, it solves the online charity supply and demand information cannot be a timely docking problem, and has the advantages of cohesion of charitable resources, such as space, and effectively promote the development of online philanthropy. From the perspective of communication technology, there are three main reasons that TikTok Live is so popular in China. First, TikTok Live is a live streaming platform with multiple fundraising technologies. Its diverse and convenient fundraising methods have greatly increased donations. Donators expect to receive real-time donation feedback. Secondly, its unique interaction method meets the social interaction needs of a wider and more diverse donation group to a certain extent; thirdly, this communication technology reduces the technical risks of online donation to a certain extent, and its unique small-amount donation and reciprocity The method of donation weakens the donor’s donation risk expectation to a certain extent and enhances the audience’s motivation to donate online. On this basis, TikTok Live has opened up a path for charitable donations and broadened the scope of donor groups and donation resources for charitable institutions. Compared with Twitch, which is a popular model for raising funds through game live broadcasts around the world, the financing technology and financing model possessed by China’s TikTok Live is a new model that is conducive to charity communication and charity financing, enriching Developed a worldwide model of charity communication and charitable donation.

However, the current "live streaming + charity" mode is still facing insufficient management, a lack of legitimacy, and an unsound regulatory system, which makes charity live streaming has become a show prop for some people or organizations to cheat traffic and money. For instance, in November 2016, the Southern Metropolis Daily revealed that the streamer of the Kuaishou live-streaming platform was faking when doing crowdfunding in Daliang Mountain, Sichuan Province, the streamer pretended to donate money to the villagers but then recovered the money after the live-streaming to get popularity and the users’ gifts. Besides, since TikTok has not made separate regulations for charity live streaming, it is categorized as general live streaming, and the money received for charity is divided by the platform. After realizing this problem, TikTok Live stipulated that such live broadcasts only allow charitable organizations that affect quality to implement charity live broadcasts on the platform and need to seek help for group projects rather than individuals. In this way, the effectiveness of "live streaming + charity" will be limited. However, according to the provisions of the Charity Law of the People’s Republic of China, individuals with charitable purposes can cooperate with charitable organizations with public fundraising qualifications, and the charitable organizations will carry out public fundraising and manage the collected funds [55]. It can be seen that the charity law only mentions public fundraising behavior, and does not negate individual help, nor does it exclude the legitimacy of such behavior. Under the background of "live streaming + charity" has become a trend, TikTok Live needs to consider the relationship between the platform’s business model and corporate social responsibility. Thus, for government departments, supervision by relevant departments should be strengthened and penalties should be increased. As for the live streaming platform, it should strengthen its own construction and create a unique platform. For users, they should actively learn the platform rules of TikTok Live, improve their media literacy, and make rational use of live streaming.

Charity live streaming as a technology to promote online charitable donations, may bring new social problems, but there is no doubt that it solves the online charity supply and demand information cannot be a timely docking problem, and has the advantages of cohesion of charitable resources, such as space, and effectively promote the development of online philanthropy. It is worth noting that the industry and academia should not regard "live streaming + charity" as a simple technology, and only focus on live streaming technology, interactive technology, or fundraising techniques. The practice of treating users as consumers rather than public benefactors, and attempting to stimulate their impulsive purchasing desire and promote their consumption intention through external factors, has deviated from the original intention of charity. As Mesthene indicated, technology is inherently neutral; it can be good or evil depending on how we decide to use it [56]. Because technology has the potential to be used for evil, it has been mistakenly labeled as so.

This paper also has certain limitations in its research. First, it only investigated TikTok Live in China, and the research results may have common methodological biases; second, the research setting Certain elements may not be sufficient. so further research should also consider experimental and longitudinal designs. For example, conduct research on the charity live broadcasts of various overseas versions of TikTok Live to expand the academic vision of charity activities on the TikTok live broadcast platform. In addition, further research could combine the social conditions and public opinions of different countries and the tradition of charitable donation to make appropriate adjustments to the various influencing factors in the model, fully explore the various influencing factors that influence donors to donate on the live broadcast platform, and then improve the model to make a useful contribution to a local charity.

## Supporting information

S1 Appendix Questionnaire. (DOCX)

S2 Appendix Variables and mesurement question. (XLS)

S1 Dataset (SAV)

S2 Dataset AMOS model test result. (AMW)
