# Supplementary material for: The influencing factor model and empirical research of TikTok charity live streaming impact users’ online charitable donation
Source: PLoS One. 2023 Nov 13;18(11):e0294186. doi: 10.1371/journal.pone.0294186 (PMC10642826; doi:10.1371/journal.pone.0294186)
Supplement: S1 Appendix — (DOCX) [file pone.0294186.s001.docx]

**Research Ethics Statement**

Dear Participants:

Thank you for participating in this study! Before you start participating in the research, please read the following ethics statement carefully to ensure that you understand the purpose of the research, the process and your rights.

1. Research purpose: The purpose of this study is to find out the situation of Tiktok Live users participating in online charitable donations through Tiktok charity live streaming. .

2. Participation is voluntary: Your participation is entirely voluntary, and you have the right to refuse to participate or withdraw from the survey at any time without any penalty or negative influence.

3. Confidentiality: Your personal information will be kept strictly confidential and only members of the research team will have access to it. In research reports, no personal information that could identify a specific individual will be disclosed.

4. Anonymity: This research is conducted anonymously and your answers will not be associated with your personal identity.

5. Data processing: The research team will analyze and interpret the collected data, and use the results for research reports and possible academic publications, but will not reveal your personal identity in any way.

6. Storage period: Your responses will be stored for a reasonable period of time and kept securely in accordance with research ethics.

7. Research results: After the study is completed, we will publish the overall results for interested participants to understand the research results.

If you have any questions or require further clarification regarding the Research Ethics Statement, please do not hesitate to contact us.

Please confirm that you have read and understood the ethics statement, and voluntarily participate in this research.

Thank you for your participation!

**Dear Participant:**

**Before you continue to participate in this survey, please confirm the following statement.**

**1. I confirm that I have read and understood this ethics statement and voluntarily participate in this questionnaire.**

**2. I understand that all responses to this survey will remain anonymous and will not be associated with my personal identity.**

**3. I understand that I have the right to refuse to participate or withdraw from the survey at any time without penalty or negative repercussions.**

**Please confirm that you have read and agree to the above statement before proceeding.**

**Survey on Participation in Online Charitable Donation through Tiktok charity live streaming**

Hello! Thank you very much for taking time out of your busy schedule to accept our questionnaire. It takes approximately 15 minutes to complete the questionnaire. The purpose of this questionnaire survey is to find out the situation of Tiktok Live users participating in online charitable donations through Tiktok charity live streaming. There is no right or wrong answer. Please answer according to your own feelings and personal actual situation. Your valuable opinions will be of great significance to the research on "Internet + Charity", Tiktok charity live streaming and online charitable donation. This questionnaire is for personal academic research, and the personal information and opinions involved will be kept strictly confidential. Sincerely thank you for your support!

**Charity live streaming** is a new form of charity communication and charitable fundraising to promote social public welfare. **On Tiktok Live, charity live streaming** refers to live broadcast activities initiated by individuals, public welfare organizations, and Tiktok platforms.

**Online charitable donation** refers to the act of donating online through network media. **On Tiktok charity live streaming room**, online charitable donation refers to charitable donations in the form of live gifting and live commerce.

**Part 1**

1. Gender

a.Male b.Female

1. Age

a.18-29 b.30-39 c.40-49 50-59 d.60 and above

1. Your monthly income level

a. less than ￥ 2,000

b. ￥2001-4000

c. ￥4001-6000

d. ￥6001-8000

e. more than ￥8000

**part 2**

1. Have you ever participated in online charitable donations through Tiktok charity live streaming?

a. Yes b. No (turn to question 8)

1. How often do you participate in online charitable donations through Tiktok charity Live streaming?

a. frequently b. occasionally c. rarely

1. In what channels do you often participate in online charitable donations through Tiktok charity live streaming?

a. live gifting b. live shopping c. both

1. What is the amount of your live gifting each time you participate in online charitable donation through Tiktok charity live streaming?
2. less than ￥10 b. ￥11-30 c. ￥31-50 d. ￥51-100 e. more than ￥100
3. What is the amount of money you spend each time you make a live commerce through Tiktok charity live streaming?
4. less than ￥200 b. ￥201-500 c. ￥501-800 d. ￥801-1000

e. more than ￥1000

1. What is your payment method for participating in online charitable donations on Tiktok charity live streaming?
2. Wechat Payment b. Alipay Payment c. Tiktok Payment d. DOU Instalment
3. Through which of the following channels did you enter the Tiktok charity live streaming?

a. Home Page Recommendation b. Same City c. Follow d. Search e. Other

1. What is the main reason why you don’t participate in online charitable donations through Tiktok charity live streaming? (Those who have participated do not answer this question)
2. I am not aware that donations can be made in this way
3. I am accustomed to traditional forms of charitable donation Inconvenient to donate
4. There's nothing I want buy or gift.
5. Concerned about the risk of the flow of funds from charitable donations made in this way.
6. Other (please fill in)

**Part3**

**Please read the following questions carefully and choose between “Strongly Agree, Agree, Unsure, Disagree, and Strongly Disagree” depending on your personal situation.**

1. **Strongly agree b. Agree c. Unsure d. Disagree e. Strongly disagree**
2. I think participating in online charitable donation through Tiktok charity live streaming has helped me in my work, study and life.
3. I think participating in online charitable donation through Tiktok charity live streaming helps to increase the effectiveness of my participation in charitable giving.
4. I think participating in online charitable donations through Tiktok charity live streaming is more intuitive and convenient than other donation methods.
5. I clearly understand how to make online charitable donations through Tiktok charity live streaming for good causes.
6. I think the process of participating in online charitable donation through Tiktok charity live streaming is clear, flexible and convenient.
7. I can skillfully make online charitable donations through Tiktok charity live streaming for good causes.
8. My friends and family or people who are important to me think I should participate in online charitable giving through Tiktok charity Live streaming.
9. My friends and family or people who are important to me participate in online charitable donation through Tiktok charity live streaming for good causes, then I will try to do the same.
10. Participating in online charitable donation through Tiktok charity live streaming for good causes can enhance my social prestige and social status.
11. Tiktok Live will provide technical support and guide me on how to make online charitable donations in the charity live streaming room.
12. Streamers will guide me on how to participate in the Tiktok charitable giving campaigns within the streams.
13. I'm concerned that making an online donation via Tiktok charity live streaming for charity will compromise my personal privacy.
14. I'm concerned about the insecurity of the payment method and flow of funds for online donations via live webcasts for good causes.
15. I'm concerned about the willingness to donate online due to the difficulty in distinguishing the authenticity of the charity programs displayed on Tiktok charity live streaming.
16. Streamers are able to answer questions from users in the comments section about the charity program and online charitable donation.
17. Streamers are able to answer users' questions about the charity programs and online charitable donation quickly.
18. Users in the comments section are able to engage in conversation around the charity project being streaming and the issue of online charitable donation.
19. I would like to participate in online charitable donation through Tiktok charity live streaming.
20. I would recommend friends, family and significant others to participate in online charitable donation through Tiktok charity live streaming.
21. I think I'll be participating in online charitable donation through Tiktok charity live streaming in the future as well.
22. I currently have an online charity donation participation in Tiktok charity live streaming.
23. I often participate in online charitable donations through Tiktok charity live streaming.
24. I recommend friends, family and significant others to participate in online charitable donation through Tiktok charity live streaming.
25. I will also be participating in online charitable donation through Tiktok charity live streaming in the future.
